# 4-Chloro-2-phenyl-2*H*-chromene-3-carbaldehyde

**DOI:** 10.1107/S2414314625006571

**Published:** 2025-07-29

**Authors:** B. Punithavalli, A. Therasa Alphonsa

**Affiliations:** a Department of Chemistry Annamalai University, Annamalai Nagar-608002 Tamilnadu India; Goethe-Universität Frankfurt, Germany

**Keywords:** crystal structure, chromene, topology

## Abstract

The title compound, 4-chloro-2-phenyl-2*H*-chromene-3-carbaldehyde (PCC), C_16_H_11_ClO_2_, was obtained in good yield by a standard one-pot method. The mol­ecule has the following substituents: a chlorine atom at the 4-position, a phenyl group at the 2-position, and an aldehyde (–CHO) group at the 3-position of the 2*H*-chromene ring system.

## Abstract 2

The title compound was obtained in good yield by a standard one-pot method. The mol­ecule has the following substituents: a chlorine atom at the 4-position, a phenyl group at the 2-position, and an aldehyde (–CHO) group at the 3-position of the 2*H*-chromene ring system.

## Graphical abstract

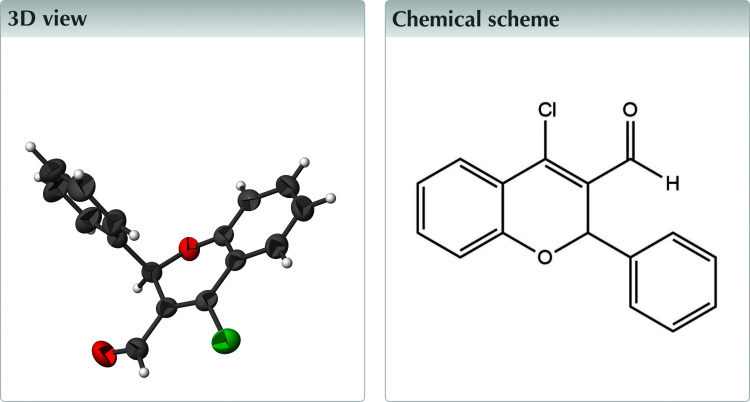

## Structure description

Heterocyclic compounds containing nitro­gen and oxygen have attracted a lot of inter­est because of their diverse pharmacological activities (Swamy & Agasimundin, 2008 ▸; Tanaka & Sugino, 2001 ▸). The basic flavonoid structure is a flavone nucleus, which consists of 15 carbon atoms arranged in rings and is available as flavones, flavonols, flavanones, isoflavones, chalcones and their derivatives. In organic chemistry, 4-chloro-2-phenyl-2*H*-chromene-3-carbaldehyde is a noteworthy compound that is especially well-known for being synthesized from flavanone. This substance is a member of the chromene class, which includes a range of physiologically active compounds with uses in drug development and medicinal chemistry. The chromene ring with a phenyl substituent and an aldehyde functional group is part of the structural framework of 4-chloro-2-phenyl-2*H*-chromene-3-carbaldehyde (PCC), which makes it useful in a variety of chemical reactions, including nucleophilic inter­actions and electrophilic substitutions (Najmanová *et al.*, 2020 ▸).

**Figure 1:**
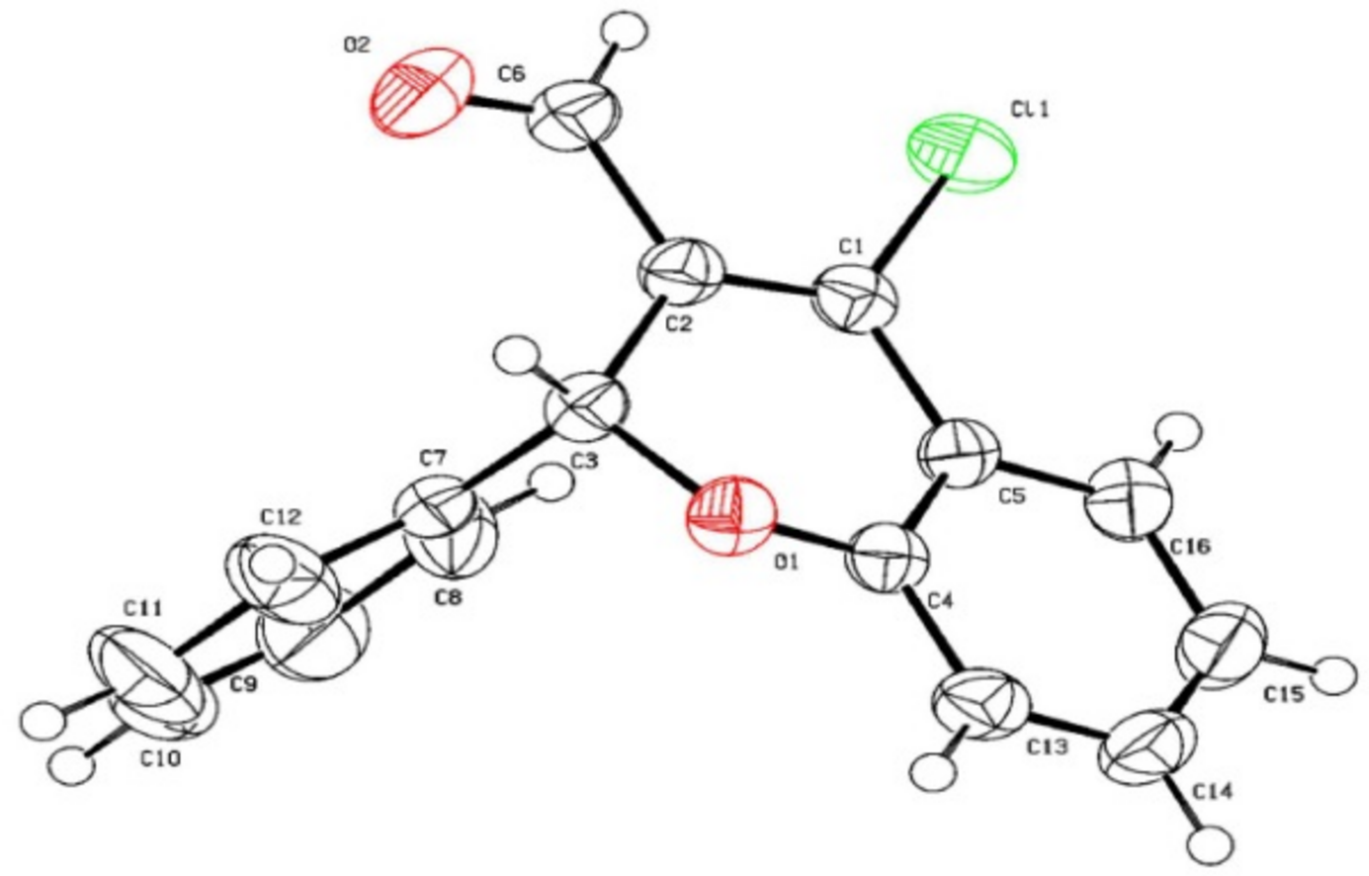
The mol­ecular structure of 4-chloro-2-phenyl-2*H*-chromene-3-carbaldehyde. Displacement ellipsoids are at the 50% probability level.

**Figure 2:**
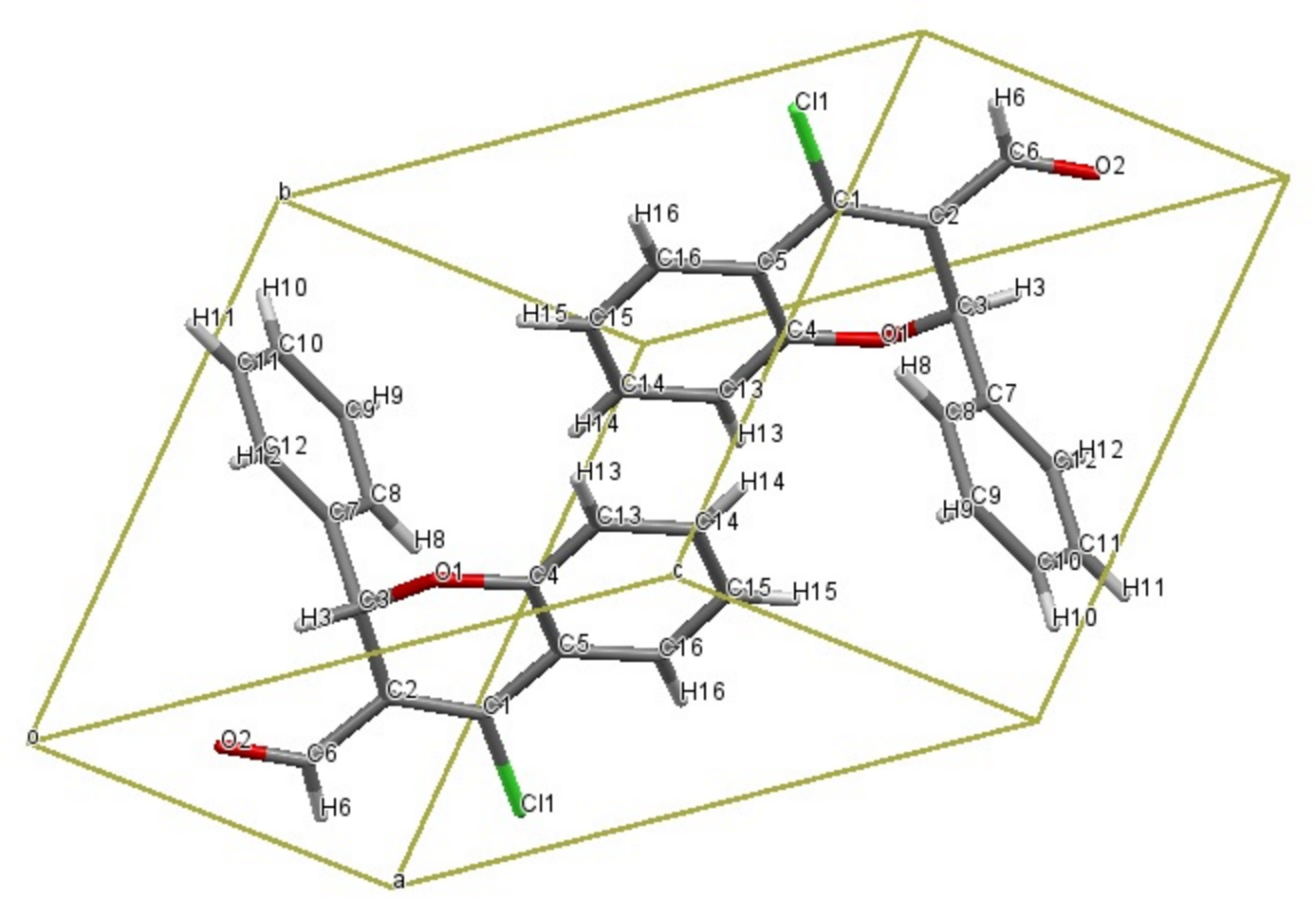
Packing of 4-chloro-2-phenyl-2*H*-chromene-3-carbaldehyde.

Single crystal X-ray analysis confirmed that the 4-chloro-2-phenyl-2*H*-chromene-3-carbaldehyde crystallizes in the triclinic system in space group *P*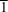. The title chromene derivative has the following substituents: a chlorine atom at the 4-position, a phenyl group at the 2-position, and an aldehyde (–CHO) group at the 3-position of the 2*H*-chromene ring system. The mol­ecular structure is shown in Fig. 1 ▸ and the unit-cell contents in Fig. 2 ▸.

## Synthesis and crystallization

**Figure 3:**
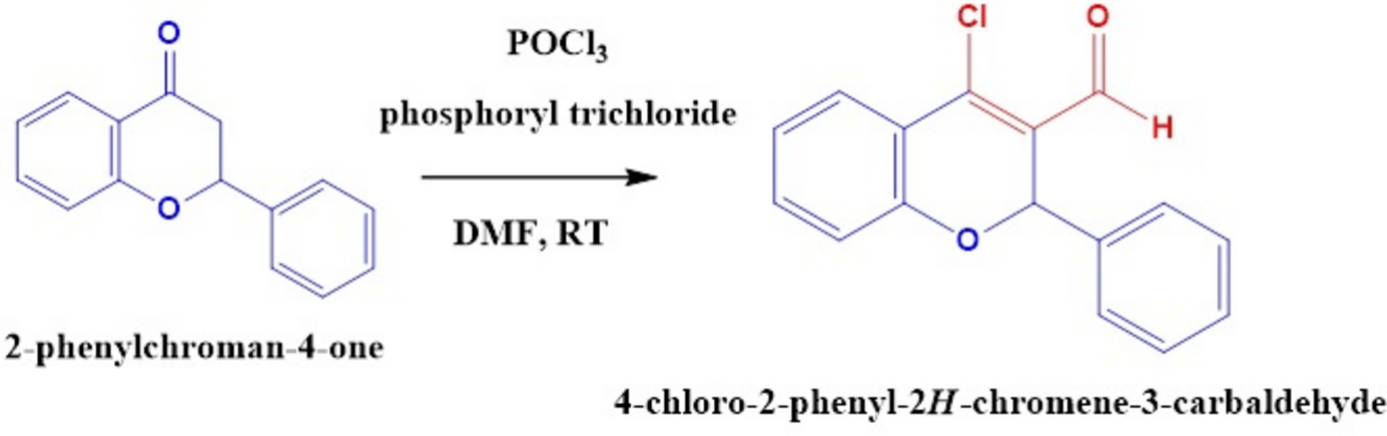
Reaction scheme for the synthesis of the title compound.

The starting materials 2-phenyl­chroman-4-one, phosphoryl trichloride and dimethyl furan were purchased from Sigma-Aldrich chemical company with a stated purity and were used as such without further purification. The title compound was synthesized according to Fig. 3 ▸. In a round-bottom flask, flavanone (0.1 g) was added with phospho­rus oxychloride (16 ml) and stirred well for 6-7 h. The completion of the reaction was monitored by TLC. After that, the reaction mixture was poured into ice-cold water. The resulting precipitate was washed with water and dried. The crude product was recrystallized from ethanol solution.

## Refinement

The crystal data and structure refinement parameters are listed in Table 1 ▸.

## Supplementary Material

Crystal structure: contains datablock(s) I, global. DOI: 10.1107/S2414314625006571/bt4178sup1.cif

Structure factors: contains datablock(s) I. DOI: 10.1107/S2414314625006571/bt4178Isup2.hkl

Supporting information file. DOI: 10.1107/S2414314625006571/bt4178Isup3.cml

CCDC reference: 2474588

Additional supporting information:  crystallographic information; 3D view; checkCIF report

## Figures and Tables

**Table 1:** Experimental details

Crystal data	
Chemical formula	C_16_H_11_ClO_2_
*M*_r_	270.70
Crystal system, space group	Triclinic, *P* 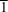
Temperature (K)	300
*a*, *b*, *c* (Å)	7.0108 (4), 8.7716 (5), 11.1285 (6)
α, β, γ (°)	75.127 (2), 83.188 (2), 73.584 (2)
*V* (Å^3^)	633.68 (6)
*Z*	2
Radiation type	Mo *K*α
μ (mm^−1^)	0.30
Crystal size (mm)	0.30 × 0.20 × 0.13

Data collection	
Diffractometer	Bruker D8 QUEST diffractometer with PHOTON II detector
Absorption correction	Multi-scan (*SADABS*; Krause *et al.*, 2015 ▸)
*T*_min_, *T*_max_	0.702, 0.746
No. of measured, independent and observed [*I* > 2σ(*I*)] reflections	24536, 3133, 2488
*R*_int_	0.039
(sin θ/λ)_max_ (Å^−1^)	0.667

Refinement	
*R*[*F*^2^ > 2σ(*F*^2^)], *wR*(*F*^2^), *S*	0.039, 0.105, 1.03
No. of reflections	3133
No. of parameters	172
H-atom treatment	H-atom parameters constrained
Δρ_max_, Δρ_min_ (e Å^−3^)	0.22, −0.21

Computer programs: *APEX4* and *SAINT* (Bruker, 2021 ▸), *SHELXT2019/1* (Sheldrick, 2015*a* ▸), *SHELXL2019/1* (Sheldrick, 2015*b* ▸) and *SHELXTL* (Sheldrick, 2008 ▸).

## full crystallographic data

### 4-Chloro-2-phenyl-2H-chromene-3-carbaldehyde Crystal data

**Table:**

C_16_H_11_ClO_2_	*Z* = 2
*M**_r_* = 270.70	*F*(000) = 280
Triclinic, *P*1	*D*_x_ = 1.419 Mg m^−^^3^
*a* = 7.0108 (4) Å	Mo *K*α radiation, λ = 0.71073 Å
*b* = 8.7716 (5) Å	Cell parameters from 9898 reflections
*c* = 11.1285 (6) Å	θ = 2.5–27.4°
α = 75.127 (2)°	µ = 0.30 mm^−^^1^
β = 83.188 (2)°	*T* = 300 K
γ = 73.584 (2)°	Block, yellow
*V* = 633.68 (6) Å^3^	0.30 × 0.20 × 0.13 mm

### 4-Chloro-2-phenyl-2H-chromene-3-carbaldehyde Data collection

**Table:**

Bruker D8 QUEST diffractometer with PHOTON II detector	2488 reflections with *I* > 2σ(*I*)
Radiation source: i-mu-s microfocus source	*R*_int_ = 0.039
φ and ω scans	θ_max_ = 28.3°, θ_min_ = 2.5°
Absorption correction: multi-scan (SADABS; Krause *et al.*, 2015)	*h* = −9→9
*T*_min_ = 0.702, *T*_max_ = 0.746	*k* = −11→11
24536 measured reflections	*l* = −14→14
3133 independent reflections	

### 4-Chloro-2-phenyl-2H-chromene-3-carbaldehyde Refinement

**Table:**

Refinement on *F*^2^	0 restraints
Least-squares matrix: full	Hydrogen site location: inferred from neighbouring sites
*R*[*F*^2^ > 2σ(*F*^2^)] = 0.039	H-atom parameters constrained
*wR*(*F*^2^) = 0.105	*w* = 1/[σ^2^(*F*_o_^2^) + (0.0403*P*)^2^ + 0.2009*P*] where *P* = (*F*_o_^2^ + 2*F*_c_^2^)/3
*S* = 1.03	(Δ/σ)_max_ < 0.001
3133 reflections	Δρ_max_ = 0.22 e Å^−^^3^
172 parameters	Δρ_min_ = −0.21 e Å^−^^3^

### 4-Chloro-2-phenyl-2H-chromene-3-carbaldehyde Special details

**Table:**

Geometry. All esds (except the esd in the dihedral angle between two l.s. planes) are estimated using the full covariance matrix. The cell esds are taken into account individually in the estimation of esds in distances, angles and torsion angles; correlations between esds in cell parameters are only used when they are defined by crystal symmetry. An approximate (isotropic) treatment of cell esds is used for estimating esds involving l.s. planes.
Refinement. Hydrogen atoms were located in a difference map and refined as riding on their parent atoms with *U*(H)=1.2*U*_eq_(C) and with C_tertiary_-H=0.98Å or with C-H=0.93Å.

### 4-Chloro-2-phenyl-2H-chromene-3-carbaldehyde Fractional atomic coordinates and isotropic or equivalent isotropic displacement parameters (Å^2^)

**Table:**

	*x*	*y*	*z*	*U*_iso_*/*U*_eq_	
Cl1	0.15627 (6)	0.98500 (5)	0.71969 (4)	0.05455 (15)	
O1	0.77862 (15)	0.76830 (13)	0.58422 (10)	0.0452 (3)	
O2	0.6807 (2)	0.95248 (17)	0.89832 (13)	0.0695 (4)	
C1	0.8078 (2)	0.60085 (18)	0.79480 (14)	0.0432 (3)	
C2	1.0062 (3)	0.5239 (2)	0.81560 (18)	0.0655 (5)	
H2	1.102219	0.579734	0.782252	0.079*	
C3	1.0636 (4)	0.3651 (3)	0.8853 (2)	0.0828 (7)	
H3	1.197400	0.315453	0.899833	0.099*	
C4	0.9247 (4)	0.2807 (2)	0.93297 (19)	0.0754 (6)	
H4	0.963825	0.173161	0.978827	0.090*	
C5	0.7274 (4)	0.3548 (2)	0.9131 (2)	0.0747 (6)	
H5	0.632676	0.297137	0.945091	0.090*	
C6	0.6681 (3)	0.5158 (2)	0.84521 (17)	0.0592 (5)	
H6	0.533599	0.566230	0.833782	0.071*	
C7	0.7540 (2)	0.77322 (18)	0.71423 (14)	0.0407 (3)	
H7	0.850791	0.827483	0.728683	0.049*	
C8	0.5509 (2)	0.87461 (17)	0.74512 (14)	0.0400 (3)	
C9	0.3969 (2)	0.88283 (17)	0.68077 (14)	0.0390 (3)	
C10	0.4248 (2)	0.80733 (17)	0.57576 (13)	0.0394 (3)	
C11	0.6219 (2)	0.74966 (17)	0.53280 (13)	0.0395 (3)	
C12	0.5371 (3)	0.9571 (2)	0.84591 (15)	0.0506 (4)	
H12	0.412489	1.015614	0.870651	0.061*	
C13	0.2713 (2)	0.7949 (2)	0.51259 (16)	0.0487 (4)	
H13	0.139474	0.833098	0.539225	0.058*	
C14	0.3141 (3)	0.7262 (2)	0.41102 (17)	0.0573 (4)	
H14	0.211223	0.717625	0.369606	0.069*	
C15	0.5087 (3)	0.6702 (2)	0.37072 (16)	0.0565 (4)	
H15	0.536306	0.624245	0.301928	0.068*	
C16	0.6630 (3)	0.68122 (19)	0.43080 (15)	0.0489 (4)	
H16	0.794096	0.642888	0.402954	0.059*	

### 4-Chloro-2-phenyl-2H-chromene-3-carbaldehyde Atomic displacement parameters (Å^2^)

**Table:**

	*U*^11^	*U*^22^	*U*^33^	*U*^12^	*U*^13^	*U*^23^
Cl1	0.0411 (2)	0.0541 (3)	0.0653 (3)	−0.00800 (17)	0.00581 (18)	−0.0173 (2)
O1	0.0397 (5)	0.0553 (6)	0.0401 (6)	−0.0157 (5)	0.0023 (4)	−0.0085 (5)
O2	0.0744 (9)	0.0788 (9)	0.0638 (8)	−0.0161 (7)	−0.0156 (7)	−0.0305 (7)
C1	0.0508 (8)	0.0403 (8)	0.0374 (7)	−0.0065 (6)	−0.0038 (6)	−0.0124 (6)
C2	0.0532 (10)	0.0599 (11)	0.0650 (12)	0.0034 (8)	0.0055 (9)	−0.0064 (9)
C3	0.0729 (14)	0.0676 (13)	0.0742 (14)	0.0201 (11)	0.0018 (11)	−0.0031 (11)
C4	0.1089 (18)	0.0433 (10)	0.0546 (11)	0.0067 (11)	−0.0072 (11)	−0.0059 (8)
C5	0.1052 (18)	0.0531 (11)	0.0657 (13)	−0.0318 (12)	−0.0130 (12)	0.0022 (9)
C6	0.0670 (11)	0.0474 (9)	0.0620 (11)	−0.0193 (8)	−0.0173 (9)	0.0004 (8)
C7	0.0415 (8)	0.0406 (8)	0.0419 (8)	−0.0128 (6)	−0.0034 (6)	−0.0098 (6)
C8	0.0449 (8)	0.0339 (7)	0.0398 (7)	−0.0111 (6)	−0.0009 (6)	−0.0057 (6)
C9	0.0386 (7)	0.0326 (7)	0.0418 (8)	−0.0099 (6)	0.0028 (6)	−0.0033 (6)
C10	0.0435 (8)	0.0342 (7)	0.0384 (7)	−0.0120 (6)	−0.0019 (6)	−0.0030 (6)
C11	0.0449 (8)	0.0341 (7)	0.0368 (7)	−0.0128 (6)	−0.0014 (6)	−0.0012 (6)
C12	0.0591 (10)	0.0465 (9)	0.0454 (9)	−0.0100 (7)	−0.0048 (7)	−0.0129 (7)
C13	0.0455 (8)	0.0484 (9)	0.0515 (9)	−0.0138 (7)	−0.0068 (7)	−0.0068 (7)
C14	0.0677 (11)	0.0553 (10)	0.0543 (10)	−0.0213 (9)	−0.0161 (8)	−0.0105 (8)
C15	0.0762 (12)	0.0515 (10)	0.0451 (9)	−0.0179 (9)	−0.0050 (8)	−0.0147 (7)
C16	0.0570 (10)	0.0440 (8)	0.0425 (8)	−0.0118 (7)	0.0034 (7)	−0.0088 (7)

### 4-Chloro-2-phenyl-2H-chromene-3-carbaldehyde Geometric parameters (Å, º)

**Table:**

Cl1—C9	1.7343 (15)	C7—C8	1.504 (2)
O1—C11	1.3641 (18)	C7—H7	0.9800
O1—C7	1.4470 (18)	C8—C9	1.341 (2)
O2—C12	1.207 (2)	C8—C12	1.464 (2)
C1—C6	1.378 (2)	C9—C10	1.453 (2)
C1—C2	1.381 (2)	C10—C13	1.397 (2)
C1—C7	1.515 (2)	C10—C11	1.402 (2)
C2—C3	1.381 (3)	C11—C16	1.381 (2)
C2—H2	0.9300	C12—H12	0.9300
C3—C4	1.364 (3)	C13—C14	1.377 (2)
C3—H3	0.9300	C13—H13	0.9300
C4—C5	1.369 (3)	C14—C15	1.376 (3)
C4—H4	0.9300	C14—H14	0.9300
C5—C6	1.390 (3)	C15—C16	1.375 (2)
C5—H5	0.9300	C15—H15	0.9300
C6—H6	0.9300	C16—H16	0.9300
			
C11—O1—C7	116.95 (11)	C9—C8—C7	118.92 (13)
C6—C1—C2	118.58 (16)	C12—C8—C7	116.38 (13)
C6—C1—C7	122.85 (15)	C8—C9—C10	121.45 (13)
C2—C1—C7	118.54 (15)	C8—C9—Cl1	121.05 (12)
C3—C2—C1	120.8 (2)	C10—C9—Cl1	117.50 (11)
C3—C2—H2	119.6	C13—C10—C11	118.49 (14)
C1—C2—H2	119.6	C13—C10—C9	124.97 (14)
C4—C3—C2	120.2 (2)	C11—C10—C9	116.48 (13)
C4—C3—H3	119.9	O1—C11—C16	117.44 (14)
C2—C3—H3	119.9	O1—C11—C10	121.73 (13)
C3—C4—C5	119.78 (19)	C16—C11—C10	120.70 (14)
C3—C4—H4	120.1	O2—C12—C8	122.83 (16)
C5—C4—H4	120.1	O2—C12—H12	118.6
C4—C5—C6	120.3 (2)	C8—C12—H12	118.6
C4—C5—H5	119.8	C14—C13—C10	120.32 (16)
C6—C5—H5	119.8	C14—C13—H13	119.8
C1—C6—C5	120.26 (19)	C10—C13—H13	119.8
C1—C6—H6	119.9	C15—C14—C13	120.12 (16)
C5—C6—H6	119.9	C15—C14—H14	119.9
O1—C7—C8	111.67 (12)	C13—C14—H14	119.9
O1—C7—C1	109.74 (12)	C16—C15—C14	120.88 (16)
C8—C7—C1	114.24 (12)	C16—C15—H15	119.6
O1—C7—H7	106.9	C14—C15—H15	119.6
C8—C7—H7	106.9	C15—C16—C11	119.49 (16)
C1—C7—H7	106.9	C15—C16—H16	120.3
C9—C8—C12	124.70 (14)	C11—C16—H16	120.3
			
C6—C1—C2—C3	0.0 (3)	C7—C8—C9—Cl1	175.11 (10)
C7—C1—C2—C3	−178.08 (18)	C8—C9—C10—C13	172.68 (14)
C1—C2—C3—C4	1.2 (3)	Cl1—C9—C10—C13	−7.6 (2)
C2—C3—C4—C5	−1.0 (3)	C8—C9—C10—C11	−10.2 (2)
C3—C4—C5—C6	−0.4 (3)	Cl1—C9—C10—C11	169.61 (10)
C2—C1—C6—C5	−1.4 (3)	C7—O1—C11—C16	−154.86 (13)
C7—C1—C6—C5	176.64 (16)	C7—O1—C11—C10	29.37 (18)
C4—C5—C6—C1	1.6 (3)	C13—C10—C11—O1	175.26 (13)
C11—O1—C7—C8	−42.03 (16)	C9—C10—C11—O1	−2.10 (19)
C11—O1—C7—C1	85.70 (15)	C13—C10—C11—C16	−0.4 (2)
C6—C1—C7—O1	−95.72 (17)	C9—C10—C11—C16	−177.74 (13)
C2—C1—C7—O1	82.32 (17)	C9—C8—C12—O2	−176.79 (16)
C6—C1—C7—C8	30.6 (2)	C7—C8—C12—O2	3.7 (2)
C2—C1—C7—C8	−151.40 (15)	C11—C10—C13—C14	0.5 (2)
O1—C7—C8—C9	30.37 (18)	C9—C10—C13—C14	177.57 (14)
C1—C7—C8—C9	−94.90 (16)	C10—C13—C14—C15	−0.4 (3)
O1—C7—C8—C12	−150.09 (13)	C13—C14—C15—C16	0.2 (3)
C1—C7—C8—C12	84.64 (16)	C14—C15—C16—C11	−0.1 (3)
C12—C8—C9—C10	175.37 (13)	O1—C11—C16—C15	−175.62 (14)
C7—C8—C9—C10	−5.1 (2)	C10—C11—C16—C15	0.2 (2)
C12—C8—C9—Cl1	−4.4 (2)		
